# The helically‐acquired CTDI_vol_ as an alternative to traditional methodology

**DOI:** 10.1002/acm2.12944

**Published:** 2020-06-09

**Authors:** Stephanie M. Leon, Robert J. Kobistek, Edmond A. Olguin, Zhongwei Zhang, Izabella L. Barreto, Bryan C. Schwarz

**Affiliations:** ^1^ Department of Radiology University of Florida College of Medicine Gainesville FL USA; ^2^ Consulting Radiological Physicist National Physics Consultants, Ltd Mentor OH USA; ^3^ Present address: Washington University School of Medicine St. Louis MO USA

**Keywords:** computed tomography, CTDI, CTDI_vol_, helical acquisition

## Abstract

**Purpose:**

Most clinical computed tomography (CT) protocols use helical scanning; however, the traditional method for CTDI_vol_ measurement replaces the helical protocol with an axial scan, which is not easily accomplished on many scanners and may lead to unmatched collimation settings and bowtie filters. This study assesses whether CTDI_vol_ can be accurately measured with a helical scan and determines the impact of pitch, collimation width, and excess scan length.

**Methods:**

CTDI_vol_ was measured for 95 helical protocols on 31 CT scanners from all major manufacturers. CTDI_vol_ was measured axially, then again helically, with the scan range set to the active area of the pencil chamber seen on the localizer image. CTDI_vol_ measurements using each method were compared to each other and to the scanner‐displayed CTDI_vol_. To test the impact of scan length, the study was repeated on four scanners, with the scan range set to the phantom borders seen on the localizer.

**Results:**

It was not possible to match the collimation width between the axial and helical modes for 12 of the 95 protocols tested. For helical and axial protocols with matched collimation, the difference between the two methods averaged below 1 mGy with a correlation of R^2^ = 0.99. The difference between the methods was not statistically significant (*P* = 0.81). The traditional method produced four measurements that differed from the displayed CTDI_vol_ by >20%; no helical measurements did. The accuracy of the helical CTDI_vol_ was independent of protocol pitch (R^2^ = 0.0) or collimation (R^2^ = 0.0). Extending the scan range to the phantom borders increased the measured CTDI_vol_ by 2.1%–9.7%.

**Conclusion:**

There was excellent agreement between the two measurement methods and to the displayed CTDI_vol_, without protocol or vendor dependence. The helical CTDI_vol_ measurement can be accomplished more easily than the axial method on many scanners and is reasonable to use for QC purposes.

## 1 INTRODUCTION

When CT systems emerged into clinical use in the 1970s, early attempts at dosimetry involved integration of the dose resulting from multiple rotations of the x‐ray tube.^1^, ^2^ The quantity measured, the multiple scan average dose (MSAD), was by definition the dose measured in a phantom from the contribution of multiple slices over an extended scan length. The reported value was the average dose in a single scan interval at the center of the scan range [Eq (1)], where the dose $D_{N,I}\left(z\right)$ is a function of position in the z‐axis, the number of scans *N*, and the scan interval *I*.^2^ Scanners at the time were quite slow, and the dosimeter used was film or an array of TLDs; thus, the MSAD was a time‐consuming and inconvenient method of CT dosimetry.

(1) $$MSAD=\frac{1}{I}\int_{-I/2}^{I/2}D_{N,I}\left(z\right)dz$$

The computed tomography dose index (CTDI) was introduced as a replacement for MSAD in 1981.^2^ Details of this methodology, which involve acquiring a single axial scan centered over a thin ionization chamber in a cylindrical acrylic phantom, are well known. The equation for CTDI is a function of the dose distribution from the single axial slice, $D\left(z\right)$ , the number of data channels used in the scan, *n*, and the width of one data channel, *T* [Eq. (2)].

(2) $$CTDI=\frac{1}{\mathrm{nT}}\int_{-\infty }^{\infty }D\left(z\right)dz$$

The relatively narrow beam widths used in early scanners allowed for most of the dose tail from this single scan to be captured by the pencil chamber, and data indicated that the ratio of the MSAD to the CTDI approached unity when a sufficient number of scans (~10) were acquired for the MSAD.^2^ Although the definition of CTDI has evolved and expanded since its inception to include $CTDI_{100},CTDI_{W}$ , and $CTDI_{\mathrm{vol}}$ ,^3^ the use of the CTDI remains the standard method of CT dosimetry to this day among regulatory agencies,^4^, ^5^ standards organizations,^6^ and accreditation bodies.^7^, ^8^

Because CTDI came to be measured with a 100‐mm pencil ionization chamber, and due to the obvious impracticalities of measuring the dose distribution at distances infinitely far from the acquired slice, the theoretical definition of CTDI is replaced by $CTDI_{100}$ in most practical applications, and the integration limits range from −50 mm to +50 mm.^3^ The meter reading (*M*) obtained in milligray (mGy) is the average air kerma over the chamber length of 100 mm; the $CTDI_{100}$ from a measurement is therefore calculated as:^3^

(3) $$CTDI_{100}\left(\text{mGy}\right)=\frac{\;\text{100 mm}\;\cdot M\text{(mGy)}}{n\cdot T\left(\text{mm}\right)}$$

The volumetric CTDI (CTDI_vol_), which yields a dose averaged over the cross‐sectional area of the phantom and is adjusted by the helical pitch, is defined as:

(4) $$CTDI_{\mathrm{vol}}\left(\mathrm{mGy}\right)=\frac{1}{p}\left(1/3\cdot CTDI_{100}^{\mathrm{center}}+2/3\cdot CTDI_{100}^{\mathrm{peripheral}}\right)$$

where *p* is the helical pitch, defined as the ratio of the table increment per tube rotation to the nominal beam width. “Center” and “peripheral” indicate the location of the chamber in the phantom. CTDI_vol_ is now the standard measurement reported by CT scanners and measured by medical physicists for routine quality control.

As previously mentioned, the standard method of measuring CTDI_vol_ requires acquiring each measurement using a single axial scan.^3^, ^7^ This methodology was developed before helical scanning was invented;^2^ however, modern scanners use helical protocols more often than axial protocols. Measuring the CTDI_vol_ from a clinical helical protocol using the traditional methodology requires “converting” it into an axial protocol, which in some scanners may lead to problems matching collimation width ( n·T ) values and bowtie filter; consequently, the tested protocol may not be a good substitute for the clinical protocol. Some nuclear hybrid units are incapable of acquiring axial scans without entering service mode, which requires the expense and inconvenience of a service call. The use of a single axial scan was implemented as a substitute for the extended scanning of MSAD for reasons that were practical in the 1980s, but the continued use of the axial scan is an anachronism that is now complicating rather than simplifying the measurement process.

Both CTDI and the older MSAD report an integrated dose that has been normalized by the scan length.^2^, ^9^ MSAD is integrated over the scan interval *I* and then divided by *I*; CTDI uses a single axial scan and divides the measurement by n·T , which is a surrogate for the beam width. Since both the MSAD and CTDI use axial scans, the dose distribution is not uniform along the z‐axis of the measurement device,^2^, ^9^ and this division by the scan interval or collimation width is logical. However, if the CTDI is measured with a helical scan instead, the individual scan planes are no longer differentiated in the x‐y plane.^9^ To calculate a normalized helical CTDI_vol_, it is necessary instead to irradiate the entire chamber, and then divide by the length of the ion chamber.

We do not suggest that the helical CTDI_vol_ as measured over the entire ion chamber is *identical* to the traditional CTDI_vol_ in the mathematical sense, but we do propose that it produces measured CTDI_vol_ values that are indistinguishable from the traditional CTDI_vol_ for all practical purposes. This study assesses whether CTDI_vol_ can be accurately measured with a helical scan and determines the impact of pitch, collimation width, and excess scan length.

It is important to appreciate the appropriate uses and limitations of CTDI_vol_. As is apparent from its name, the CTDI_vol_ is a dose *index* intended for system comparisons and quality control purposes. It is not a patient dose, although attempts have been made to use it to approximate patient dose.^10^, ^11^ Additionally, there are known problems with its application to modern CT scanners that employ wide beams and scan modes without table movement.^6^ Proposals to update the measurement methodology have not been widely adopted due largely to the requirement for new test equipment, in particular the recommendation to use much longer phantoms.^6^ This study does not attempt to address the theoretical limitations of CTDI_vol_, nor make it more accurately reflect a true patient dose; the focus is on updating the measurement methodology for implementation in modern scanners.

## 2 MATERIALS AND METHODS

### 2.A Helical CTDI formalism

The traditional axially‐acquired volumetric CTDI will be denoted $CTDI_{\mathrm{vol}}^{A}$ and the helically‐acquired volumetric CTDI will be denoted $CTDI_{\mathrm{vol}}^{H}$ . The calculation of $CTDI_{\mathrm{vol}}^{H}$ requires a minor modification to the traditional CTDI equation. For a helical acquisition covering the entire pencil chamber and measured in mGy, Eq. 4 is replaced by Eq. (5):

(5) $$CTDI_{\mathrm{vol}}^{H}\left(\mathrm{mGy}\right)=\left(1/3\cdot M_{H}^{\mathrm{center}}+2/3\cdot M_{H}^{\mathrm{peripheral}}\right)$$

where *M_H_* is the meter reading from the helical acquisition. Thus, the final calculation of $CTDI_{\mathrm{vol}}^{H}$ is actually independent of *n, T, p*, and the length of the ion chamber. While this calculation may seem nonintuitive, it can be understood by considering that the dose distribution from the helical acquisition is approximately flat across the length of the ion chamber,^9^ and therefore we can assign a value for “collimation width” of n·T=100mm . It is unnecessary to correct for pitch because the meter reading was acquired with the clinical pitch applied.

The formalism of denoting the axially‐acquired CTDI_vol_ with a superscript *A* and the helically‐acquired CTDI_vol_ with a superscript *H* is adopted here for brevity when referring to them in the subsequent sections of this paper.

### 2.B Comparison of ${\text{CTDI}}_{\text{vol}}^{A}{\;\text{and CTDI}}_{\text{vol}}^{H}$

A total of 31 CT scanners and 95 protocols were tested. Scanners of all major manufacturers were included: 10 GE, 5 Philips, 6 Siemens, and 10 Canon. The scanners included models ranging from 16 to 320 slices and were manufactured between 2005 and 2017. The clinical protocols specified by the ACR for routine quality control (adult head, adult abdomen, pediatric head, and pediatric abdomen) were tested for all scanners in which these protocols were helical and used clinically. Features of the clinical protocols including the collimation width and pitch were recorded for further analysis. For each scan protocol tested, $CTDI_{\mathrm{vol}}^{A}$ was measured and followed by $CTDI_{\mathrm{vol}}^{H}$ . The scanner‐calculated CTDI_vol_ was recorded from the protocol planning page prior to scanning.

$CTDI_{\mathrm{vol}}^{A}$ was measured using the methodology described by the American College of Radiology (ACR) and calculated using Eqs. (3) and (4).^7^ If the collimation width (defined as the product n·T ) of the clinical helical scan could not be matched in axial mode, the closest available collimation width was used, per ACR recommendations. Bowtie filters were matched in most, and possibly all, cases, although it was not possible to verify the bowtie filter used for all scanners. Because many measurements were acquired in the consulting environment during routine annual inspections, verification of collimation width and bowtie filter matching was done from the user interface rather than the image DICOM headers.

**Fig. 1:**
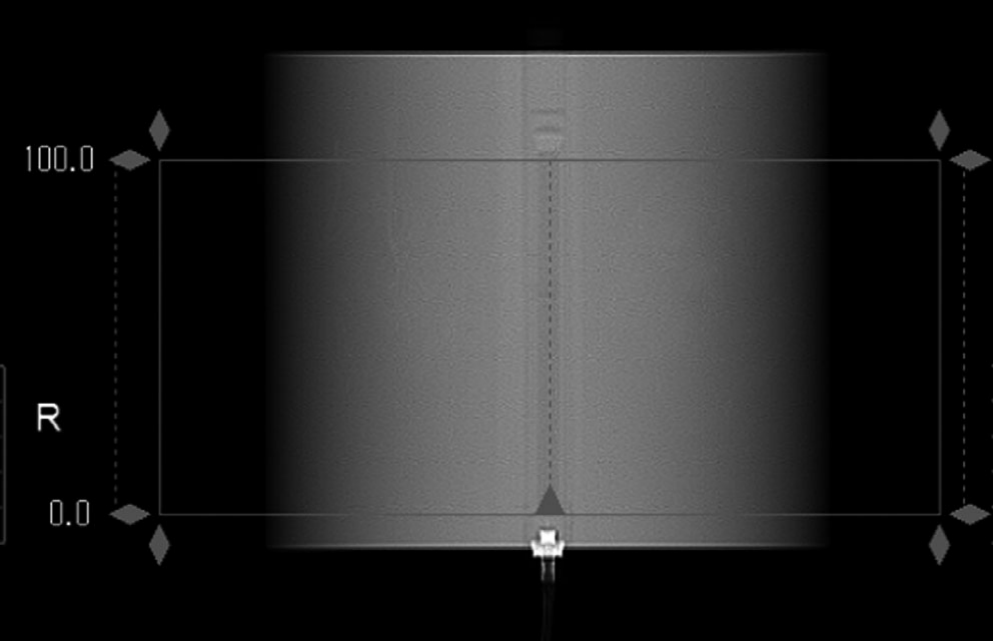
Topogram of the computed tomography dose index phantom with pencil chamber inserted. The air volume of the chamber is used to set the scan range.

The measurement of $CTDI_{\mathrm{vol}}^{H}$ was acquired as follows:

- A localizer image of the phantom with pencil chamber inserted into the appropriate CTDI phantom was acquired.
- The clinical protocol was selected. If the protocol utilized mA modulation, it was turned off and a clinically appropriate manual mA was selected, but otherwise the clinical protocol was not altered.
- The scan length was set equal to the active length of the chamber visible on the localizer (Fig. 1). The scan length was verified to be 100 mm and adjusted manually if necessary.
- Exposure was measured three times in the center phantom hole and three times in the 12:00 peripheral hole. The CTDI_vol_ was calculated from the measurements using Eq. (5).

Reproducibility of the $CTDI_{\mathrm{vol}}^{A}$ and $CTDI_{\mathrm{vol}}^{H}$ measurements was assessed by completing the entire measurement procedure five times consecutively for the adult head protocol and the adult abdomen protocol on three different scanners: one GE, one Siemens, and one Canon. The coefficients of variation (CV) measured were found to be similar for the three scanners, for a given phantom size and measurement method, so the average CV was used to estimate the error in all scans.

Data analyses were performed to assess, for each protocol tested:

- The absolute and percent differences between $CTDI_{\mathrm{vol}}^{A}$ and $CTDI_{\mathrm{vol}}^{H}$
- Statistical analysis of the methodologies by orthogonal linear regression and a two‐tailed t‐test for paired samples, using JMP Pro^1^ statistical software
- Agreement between the methodologies as a function of collimation width
- Agreement between the methodologies as a function of helical pitch.

The absolute and percent differences between the axial and helical measurements were also compared to the scanner‐displayed CTDI_vol_. These scanner‐displayed values have a very loose tolerance for accuracy (±20% according to accreditation agencies^8^, ^12^ and as much as ±40% according to some manufacturer specifications^13^, ^14^); thus, they are not considered to be the ground truth against which the accuracy of each measurement method is compared. However, it is important to know if implementation of the helical method is likely to result in worse agreement with the manufacturer's reported value, as this may complicate adoption of the helical methodology.

### 2.C Investigation of the impact of excess scan length

On some localizer images, particularly using the large phantom, it can be difficult to see the air volume of the pencil chamber. Although this can be overcome with sufficient adjustment of the window width and level, it would be easier to simply scan the entire phantom when measuring $CTDI_{\mathrm{vol}}^{H}$ . Scanning the entire phantom would also make the procedure less prone to errors that may be introduced by unintended shifts in the position of the ion chamber. For one scanner from each manufacturer (a total of four scanners), the entire procedure was repeated with the scan range set to the phantom borders visualized on the localizer, rather than the chamber air volume. Thus, 16 protocols, 4 from each manufacturer, were assessed. The percent difference and absolute difference in measured $CTDI_{\mathrm{vol}}^{H}$ between the full‐phantom and chamber‐only scan ranges were calculated.

## 3 RESULTS

It was not possible to match the collimation width between the axial and helical modes for 12 of the 95 protocols tested (12.6%). When directly comparing the measurements of $CTDI_{\mathrm{vol}}^{A}$ to $CTDI_{\mathrm{vol}}^{H}$ , or $CTDI_{\mathrm{vol}}^{A}$ to the scanner display from the helical protocol, these protocols have been excluded in some cases. Data should be assumed to include *all* measurements unless specifically labeled “matched collimation.”

Reproducibility measurements (Table 1) indicate that the CVs of the CTDI_vol_ measurements for the body phantom in helical mode and the head phantom in both axial and helical modes were all very similar, ranging from 0.1 to 0.4%. The average CV for the body phantom in axial mode ranged from 1 to 4%.

**Table 1:** Reproducibility statistics from five consecutive scans for each combination of phantom size and manufacturer.

Phantom	Manufacturer	Axial CTDI			Helical CTDI		
		Mean	SD	CV	Mean	SD	CV
Head	Canon	39.7	0.061	0.15%	37.0	0.064	0.17%
	GE	53.9	0.228	0.42%	55.0	0.168	0.30%
	Siemens	46.3	0.018	0.04%	43.4	0.038	0.09%
Body	Canon	14.2	0.187	1.31%	15.1	0.028	0.19%
	GE	15.0	0.381	2.55%	14.9	0.032	0.22%
	Siemens	8.9	0.372	4.17%	8.2	0.026	0.32%

**Fig. 2:**
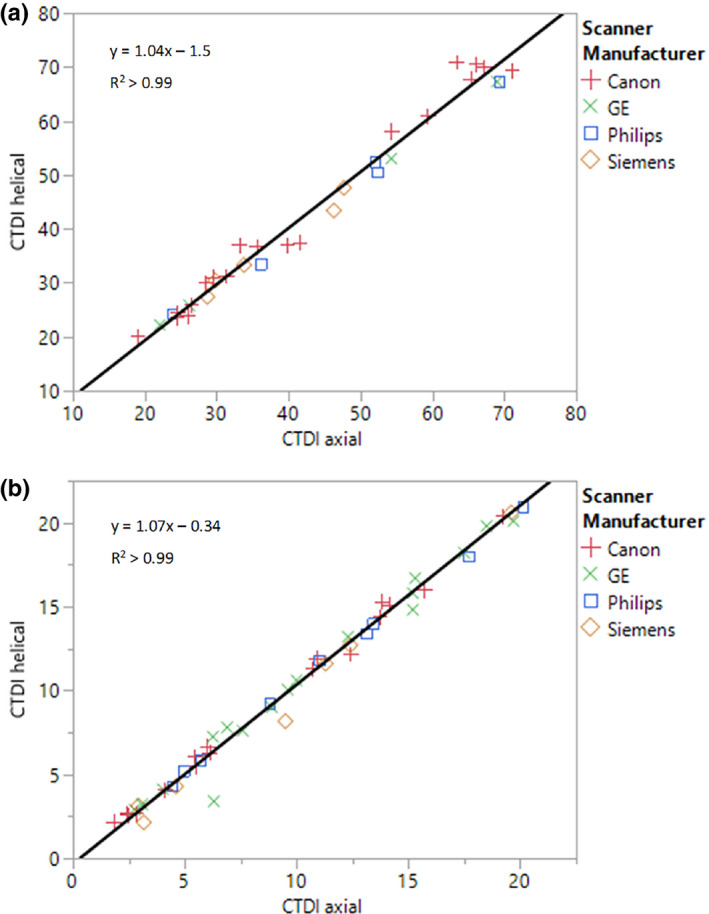
Linear regression for scans with matched collimation using (a) the head phantom and (b) the body phantom.

**Fig. 3:**
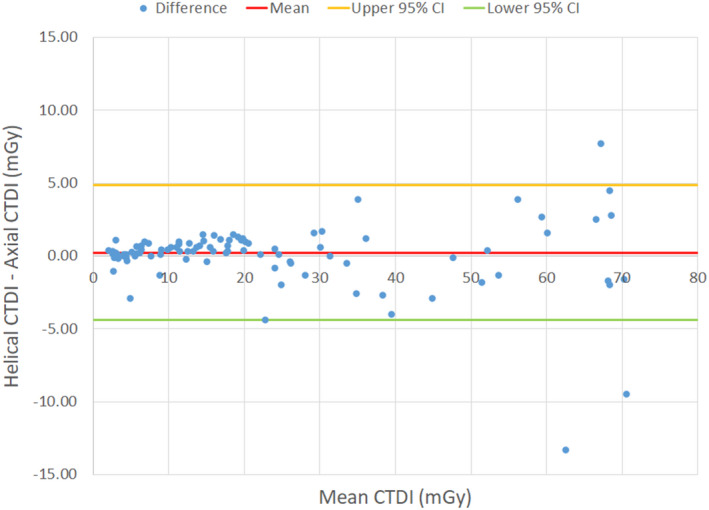
Bland–Altman plot showing differences between the helical and axial computed tomography dose index measurement methods.

Table 2 summarizes the absolute differences between $CTDI_{\mathrm{vol}}^{A}$ and the scanner display; $CTDI_{\mathrm{vol}}^{H}$ and the scanner display; and $CTDI_{\mathrm{vol}}^{A}$ and $CTDI_{\mathrm{vol}}^{H}$ . The average differences were less than 1 mGy for all protocols. Across all protocols, the difference between $CTDI_{\mathrm{vol}}^{A}$ and $CTDI_{\mathrm{vol}}^{H}$ averaged only 0.3 mGy. Figures 2(a) and 2(b) show the results of the orthogonal linear regression using the head and body phantoms, respectively. The analyses were separated by phantom size to account for the different variance ratios obtained from the reproducibility measurements. The head phantom data were fit with a slope of 1.04 and intercept of −1.5. The body phantom data were fit with a slope of 1.07 and intercept of −0.34. The correlation coefficient for both fits was greater than 0.99. A Bland–Altman plot further analyzing the differences between $CTDI_{\mathrm{vol}}^{A}$ and $CTDI_{\mathrm{vol}}^{H}$ shows excellent agreement between the measurements, with a 95% confidence interval of −4.4 mGy to +4.9 mGy (Fig. 3). The *P*‐value resulting from the paired *t*‐test was 0.81, indicating no significant difference between the two methodologies.

**Table 2:** Average differences in mGy between axial CTDI_vol_ measurements, helical CTDI_vol_ measurements, and displayed CTDI_vol_ values, with 95% confidence intervals. ^a^Matched collimation only.

Protocol	Average displayed CTDI (mGy)	Difference between axial and displayed CTDI (mGy)^a^	Difference between helical and displayed CTDI (mGy)	Difference between helical and axial CTDI (mGy)^a^
Adult head	57.4	−0.1 (−11.1, 10.8)	−0.6 (−9.3, 8.2)	0.4 (−5.7, 6.6)
Adult abdomen	14.2	0.0 (−2.4, 2.5)	0.5 (−2.1, 3.1)	0.6 (−0.6, 1.7)
Pediatric head	27.4	−0.1 (−5.6, 5.4)	−0.1 (−4.3, 4.1)	0.1 (−2.9, 3.1)
Pediatric abdomen	4.6	0.1 (−1.7, 1.9)	0.1 (−0.8, 1.0)	0.0 (−1.5, 1.5)

**Fig. 4:**
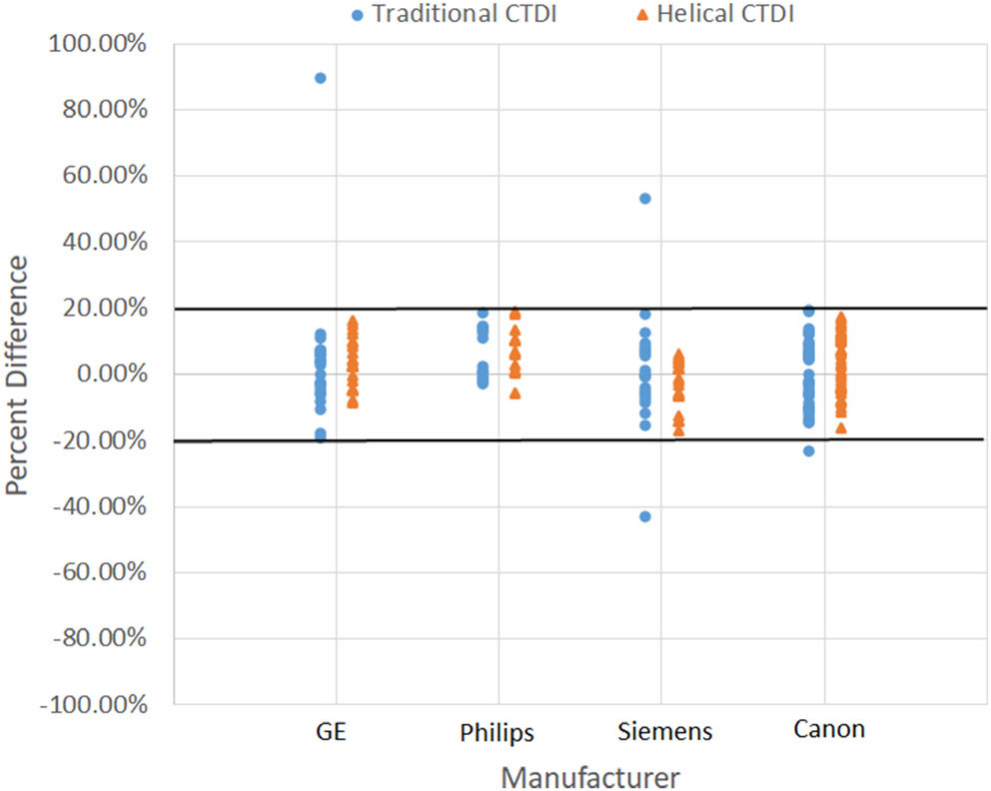
Percent difference between measured and scanner‐displayed CTDI_vol_ values. The bold line marks the American College of Radiology and TJC limit of 20%.

The percent differences between each methodology and the scanner display are shown in Fig. 4. This agreement is of importance because both the ACR and The Joint Commission specify a maximum difference of 20% between the measurement and the scanner display, and the displayed CTDI_vol_ value may be used for patient dose estimates, dose monitoring, or dose alerts. The $CTDI_{\mathrm{vol}}^{H}$ agrees with the display as well as or better than the $CTDI_{\mathrm{vol}}^{A}$ , regardless of manufacturer. Four protocols had discrepancies of >20% from the display when measuring the $CTDI_{\mathrm{vol}}^{A}$ , but all of these discrepancies dropped to <20% when using the $CTDI_{\mathrm{vol}}^{H}$ . Of these four protocols, one Siemens protocol had unmatched collimation and unverified matching of the bowtie filter. The other three protocols were matched for both collimation width and bowtie filter.

**Fig. 5:**
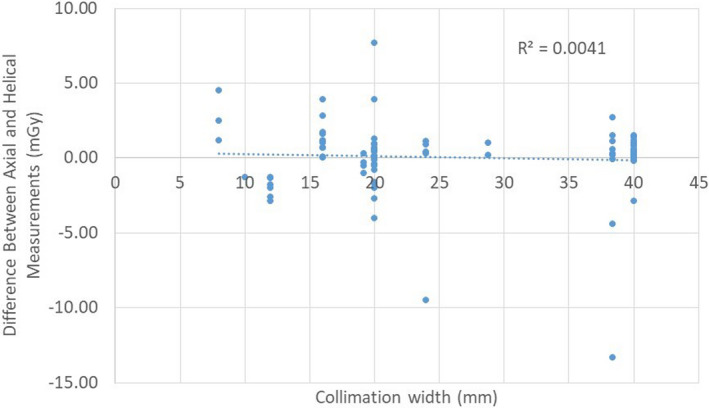
Differences between axial and helical measurements as a function of collimation width.

**Fig. 6:**
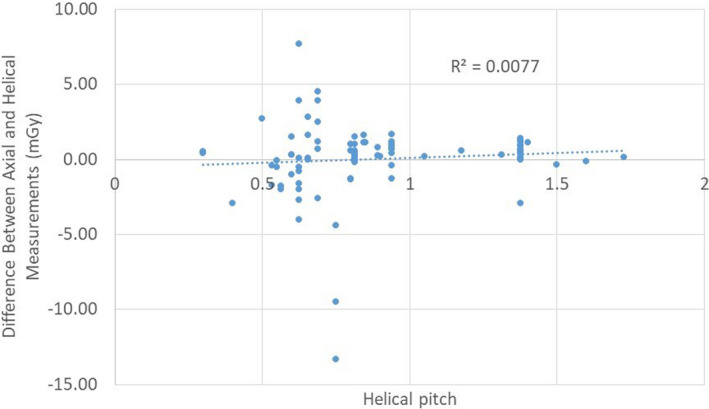
Differences between axial and helical measurements as a function of helical pitch.

The collimation widths tested ranged from 8 to 40 mm. The pitches ranged from 0.298 to 1.728. The collimation widths and pitches evaluated were those used in the clinical protocols, and do not necessarily represent the entire range available on the scanners. A linear regression of the difference in mGy as a function of collimation width produced a correlation coefficient of 0.0 (Fig. 5); a linear regression as a function of pitch also resulted in a correlation coefficient of 0.0 (Fig. 6). These linear regressions demonstrate that the difference between $CTDI_{\mathrm{vol}}^{A}$ and $CTDI_{\mathrm{vol}}^{H}$ was not dependent on either collimation width or pitch.

Setting the scan range long enough to scan the entire phantom, rather than just the 100‐mm length of the pencil chamber, increased the measured $CTDI_{\mathrm{vol}}^{H}$ in all cases (Table 3). This increase averaged 5.7% (range 2.1%–9.7%), and was statistically significant with a *P* value of 0.0007. The agreement with $CTDI_{\mathrm{vol}}^{A}$ was better using the longer scan range in nine of the 16 protocols tested (2 Canon, 1 GE, 2 Philips, and all 4 Siemens).

**Table 3:** Measurements acquired for one scanner of each major manufacturer using the axial method, the helical method with the scan range equal to the chamber length, and the helical method with the scan range equal to the full‐phantom length. All scanners were able to match collimation and bowtie filter between the axial and helical protocols.

	Scanner‐displayed CTDI (mGy)	Axial CTDI (mGy)	Chamber‐only helical CTDI (mGy)	Full‐phantom helical CTDI (mGy)	% Diff between axial and chamber only	% Diff between axial and full phantom	% Diff between chamber‐only and full phantom
Canon							
Adult head	34.90	39.7	37.0	39.0	−6.8%	−1.6%	5.6%
Adult abdomen	13.1	14.1	15.1	15.5	7.3%	9.6%	2.1%
Pediatric head	22.9	25.9	23.9	25.3	−7.7%	−2.3%	5.8%
Pediatric abdomen	2.3	2.4	2.6	2.7	6.3%	9.3%	2.8%
GE							
Adult head	49.70	53.9	55.0	58.7	2.1%	8.9%	6.7%
Adult abdomen	14.17	15.2	14.8	15.5	−2.5%	2.2%	4.8%
Pediatric head	11.33	11.8	11.9	12.7	0.6%	7.2%	6.5%
Pediatric abdomen	4.02	4.0	4.1	4.2	2.1%	5.4%	3.2%
Philips							
Adult head	45.70	52.3	50.5	53.3	−3.6%	1.8%	5.6%
Adult abdomen	17.6	20.1	21.0	21.4	4.2%	6.5%	2.2%
Pediatric head	30.4	36.1	33.5	35.9	−7.1%	−0.5%	7.1%
Pediatric abdomen	7.8	8.8	9.2	9.5	4.9%	8.6%	3.5%
Siemens							
Adult head	42.84	46.3	43.4	47.6	−6.2%	2.8%	9.7%
Adult abdomen	8.03	8.9	8.2	8.9	−8.5%	0.2%	9.4%
Pediatric head	26.27	28.7	27.4	29.5	−4.6%	2.7%	7.6%
Pediatric abdomen	2.07	3.2	2.1	2.3	−32.4%	−27.1%	8.0%

## 4 DISCUSSION

Although it is not a perfect metric, CTDI_vol_ has long been the standard dose measurement in CT. It is displayed by all modern scanners and is enshrined in regulations, industry standards, and accreditation documents. Despite its detractors, it is unlikely to be replaced in the near future. However, using a single axial scan to measure it is difficult on some modern scanners, and may lead to measurements which do not accurately reflect the clinical protocol being tested due to mismatched collimation width or bowtie filter. Of the scanners tested, three of the four manufacturers had at least one helical protocol with a collimation width that could not be matched by the user in an axial acquisition mode, so the issue is fairly widespread. Ensuring a match of the bowtie filter is even more problematic, since the filter used in a given protocol is often not visible to the user. The proposed measurement methodology avoids these complications by using the clinical protocol with a minimum of alteration.

The current study clearly shows that CTDI_vol_ can be measured with a helical acquisition; the correlation to the axially acquired CTDI_vol_ is nearly perfect, and the average difference between the resulting values is statistically insignificant. While we do not suggest that the helical CTDI_vol_ is identical to the traditional CTDI_vol_ in the mathematical sense, the evidence presented here suggests that the CTDI_vol_ measurements produced by the helical method are indistinguishable from those produced by the traditional method for clinical purposes. That the agreement between the two methods shows no dependence on pitch or collimation width provides further confidence that $CTDI_{\mathrm{vol}}^{H}$ is an acceptable substitute for $CTDI_{\mathrm{vol}}^{A}$ .

There are benefits to the helical acquisition of CTDI_vol_ besides the simple accommodation of scanners that cannot easily produce axial protocols equivalent to their helical counterparts. The first is that the peripheral measurements from a single axial slice, particularly in the body phantom, are prone to variation; in thus study, the CV of the of CTDI_vol_ was found to be an order of magnitude higher using the axial scan than using the helical scan. This increased CV is the result of increased variance in the peripheral $CTDI_{100}^{A}$ reading, a known consequence of variability in tube start location and beam overlap at the start and end position of tube rotation.^15^ This overlap is of consequence only for the case of an axial acquisition; variability in tube start and stop positions in the helical scan contributes little to the integrated exposure from many rotations. Thus, the $CTDI_{\mathrm{vol}}^{H}$ displays less measurement variability using the body phantom than does the traditional $CTDI_{\mathrm{vol}}^{A}$ .

A secondary possible benefit to the helical acquisition is that the resulting measurements appear less prone to large discrepancies from the manufacturer‐reported CTDI_vol_ values. Although the scanner‐displayed values should not to be taken as an accurate ground truth, equal‐or‐better agreement is convenient for accreditation testing. In this study, the $CTDI_{\mathrm{vol}}^{A}$ measured from four protocols differed from the displayed CTDI_vol_ value by more than 20%, which would typically be reported as a failure that would result in a service call to “fix” the displayed value. However, all large discrepancies disappeared when $CTDI_{\mathrm{vol}}^{H}$ was used instead. It should be noted that all four failures were from protocols with relatively low displayed CTDI_vol_ values (2.07, 3.32, 2.07, and 14.2 mGy); thus, large percent differences were observed despite the absolute differences all being less than about 3 mGy. It is possible that the improved agreement was due to errors in reproducibility of the axial measurements, as described in the previous paragraph. Another possible explanation for the improvement is the possibility that some of the bowtie filters remained unmatched between the axial and helical protocols, despite efforts to match them (thus highlighting one of the motivations for using the helical clinical protocol for testing). It is also possible that the manufacturers use a helical technique to produce their displayed values, thus leading the $CTDI_{\mathrm{vol}}^{H}$ to be slightly closer to the calibrated values.

When implementing the helical methodology, the geometric accuracy of the localizer image should be considered, since the localizer is used to set the scan range over the pencil chamber. Testing the localizer accuracy, which is an annual test already required by the ACR, is recommended prior to using the helical methodology.

The answer to whether it is acceptable to scan the entire phantom rather than just the chamber is not clear‐cut. Scanning the entire phantom does bias the measurements upwards by up to 10% due to the increased scatter detected, which stems both from the additional scan length as well as differences in adaptive z‐axis collimation at the ends of the ion chamber. However, the intercepts of the orthogonal linear regressions (Fig. 2) were both slightly negative, so this bias may slightly improve the agreement with $CTDI_{\mathrm{vol}}^{A}$ . By including the additional scatter contribution from the longer scan length, the $CTDI_{\mathrm{vol}}^{H}$ acquired over the entire phantom more closely approximates the theoretical definition of CTDI [Eq. (2)]. However, the agreement with the manufacturer display will likely suffer, as one can imagine by mentally shifting the orange markers up in Fig. 4. At this time, we would recommend adherence to the chamber‐only protocol since it is more thoroughly tested, produces good agreement with the traditional axial measurements, and has good agreement with the manufacturer‐displayed CTDI_vol_. Extension of the scan range by a few millimeters beyond the air volume of the ion chamber can help avoid errors due to small movements of the chamber or difficulty perceiving the air volume clearly, with minimal impact on the measured CTDI_vol_. Extending the scan range to the edges of the phantom is not generally necessary.

There are several limitations to this work. Because this work focused on the four clinical protocols that are tested for ACR accreditation, collimation widths in excess of 40 mm were not tested. When wider beams are available, they are typically used for specialized studies that are not tested for accreditation purposes. Other specialized studies, such as those using multi‐energy protocols, were likewise outside the scope of the study. This is not to imply that the dose from these studies is not of interest; however, in the interest of being able to validate the method for a wide variety of clinical scanners, testing was limited to protocols that were included as part of routine testing. The possible effect of adaptive collimation was not considered in this work, either.

Another limitation is the possibility that not all bowtie filters were matched between the axial and helical scans. The authors believe that all or almost all were matched; however, because many of the measurements were acquired in the consulting environment, the DICOM header information was not obtained to verify postscan.

Other limitations apply to the comparison of the measured CTDI_vol_ and the displayed CTDI_vol_. First, the way that manufacturers calculate CTDI_vol_ is not entirely standardized, and some manufacturer manuals demonstrate a dependence on z‐coverage and pitch that is incorrect according to IEC standard 60601‐2‐44.^16^ As mentioned previously, the displayed CTDI_vol_ should not be used as the gold standard against which the measurement is compared, but it is possible that the good agreement to the displayed value found in this study would not apply to all protocols or all scanners due to these variances. Second, the use of the prescan CTDI_vol_ estimate rather than the post‐scan value is a limitation to the accuracy of the comparison between the displayed value and the measured values. While these values are typically very similar when scanning with a fixed technique, they are not always identical.

## 5 CONCLUSIONS

There was excellent agreement between the axial and helical CTDI_vol_ measurement methods, with a correlation of >0.99 and no statistically significant difference observed. CTDI_vol_ measurements produced with the helical method also agreed well with the CTDI_vol_ displayed by the scanner. Only the axial mode measurements produced large discrepancies compared to the display.

The helical CTDI_vol_ measurement does not depend on helical pitch or collimation width and can be accomplished more easily than the axial method on many scanners. The measurement of CTDI_vol_ with a helical acquisition is a reasonable alternative to the traditional axial method for QC purposes.

## CONFLICT OF INTEREST

No conflict of interest.

## Note

^1^ JMP Pro version 14.1.0, SAS Institute Inc., 100 SAS Campus Drive, Cary, NC 27513.

## References

[1] Bauhs JA , Vrieze TJ , Primak AN , Bruesewitz MR , McCollough CH . CT dosimetry: comparison of measurement techniques and devices. Radiographics. 2008;28:245–253. 18203941 10.1148/rg.281075024

[2] Shope TB , Gagne RM , Johnson GC . A method for describing the doses delivered by transmission x‐ray computed tomography. Med Phys. 1981;8:488–495. 7322067 10.1118/1.594995

[3] AAPM Report No. 96: The Measurement, Reporting, and Management of Radiation Dose in CT. College Park, MD: American Association of Physicists in Medicine; 2007.

[4] Performance Standards for Ionizing Radiation Emitting Products. In: FDA, ed. *21 C.F.R. 1020.33*. Rev. April 1, 2018 ed.

[5] Provision for alternate measure of the computed tomography dose index (CTDI) to assure compliance with the dose information requirements of the federal performance standard for computed tomography. FDA Center for Devices and Radiological Health; 2006.

[6] AAPM Report No. 111: Comprehensive methodology for the evaluation of radiation dose in x‐ray computed tomography. College Park, MD: American Association of Physicists in Medicine; 2010.

[7] American College of Radiology CT Accreditation Program Testing Instructions. Rev. 10/10/18 ed: American College of Radiology; 2018.

[8] Diagnostic Imaging Requirements Applicable to Ambulatory Care Centers. The Joint Commission; 2015.

[9] Anam C , Haryanto F , Widita R , Arif I , Dougherty G . Profile of CT scan output dose in axial and helical modes using convolution. J Phys: Conf Ser. 2015;694:012034.

[10] AAPM Report No. 204: Size‐Specific Dose Estimates (SSDE) in Pediatric and Adult Body CT Examinations; 2011.

[11] AAPM Report No. 220: Use of Water Equivalent Diameter for Calculating Patient Size and Size‐Specific Dose Estimates (SSDE) in CT; 2014. PMC4991550 27546949

[12] American College of Radiology CT Accreditation Program Testing Instructions Rev. 10/10/18. American College of Radiology; 2018.

[13] Revolution™ CT technical reference manual direction 5443887‐1EN, revision 3. GE Healthcare; 2015.

[14] SOMATOM Force: System Owner Manual ‐ Dosimetry and imaging performance report. SIEMENS Healthineers.

[15] Horie M , Ursani A , Mehrez H , Paul N . Pitfalls in measurement of peripheral CT radiation dose and effect on patient skin dose. Paper presented at: European Congress of Radiology 2012; Vienna, Austria.

[16] Medical Electrical Equipment ‐ Part 2‐44: Particular Requirements For The Basic Safety And Essential Performance Of X‐Ray Equipment For Computed Tomography. IEC 60601‐2‐44 Ed. 3.2 ed: International Electrotechnical Commission; 2016.

